# Wolbachia Infection Associated with Increased Recombination in *Drosophila*

**DOI:** 10.1534/g3.118.200827

**Published:** 2018-11-20

**Authors:** Nadia D. Singh

**Affiliations:** Department of Biology, University of Oregon Eugene OR, 97403

**Keywords:** Recombination, phenotypic plasticity, *Drosophila*, Wolbachia

## Abstract

Wolbachia is a maternally-transmitted endosymbiotic bacteria that infects a large diversity of arthropod and nematode hosts. Some strains of Wolbachia are parasitic, manipulating host reproduction to benefit themselves, while other strains of Wolbachia exhibit obligate or facultative mutualisms with their host. The effects of Wolbachia on its host are many, though primarily relate to host immune and reproductive function. Here we test the hypothesis that Wolbachia infection alters the frequency of homologous recombination during meiosis. We use *D. melanogaster* as a model system, and survey recombination in eight wild-derived Wolbachia-infected (strain *wMel)* and Wolbachia-uninfected strains, controlling for genotype. We measure recombination in two intervals of the genome. Our results indicate that Wolbachia infection is associated with increased recombination in one genomic interval and not the other. The effect of Wolbachia infection on recombination is thus heterogenous across the genome. Our data also indicate a reproductive benefit of Wolbachia infection; infected females show higher fecundity than their uninfected genotypic controls. Given the prevalence of Wolbachia infection in natural populations, our findings suggest that Wolbachia infection is likely to contribute to recombination rate and fecundity variation among individuals in nature.

Wolbachia is genus of Gram-negative bacteria that infects a wide variety of arthropod and nematode host species . As many as 40–66% of arthropods are infected with Wolbachia (Hilgenboecker *et al.* 2008; Zug and Hammerstein 2012) and in many cases, Wolbachia infection is observed at high frequencies in natural populations (*e.g.*, Turelli and Hoffmann 1995). Wolbachia occupy the range of the parasite-mutualist spectrum in arthropods; there are myriad examples of Wolbachia behaving as a reproductive parasite as well as Wolbachia conferring fitness benefits to the host (for review see Zug and Hammerstein 2015). With respect to the former, Wolbachia have been shown to induce cytoplasmic incompatibility (*e.g.*, Breeuwer and Werren 1990; O’neill and Karr 1990; Mercot *et al.* 1995; Perrot-Minnot *et al.* 1996) and parthenogenesis (for review see Huigens and Stouthamer 2003; Engelstädter and Hurst 2009). Wolbachia infection also can manipulate its host to produce female-biased sex ratios in other ways including feminization and male-killing (for review see Kageyama *et al.* 2012).

In Drosophila, strains of Wolbachia span the parasite-mutualist spectrum as well. Male-killing strains of Wolbachia are present in a number of Drosophila species (Hurst *et al.* 2000; Jaenike *et al.* 2003; Sheeley and Mcallister 2009; Richardson *et al.* 2016), as are strains that induce cytoplasmic incompatibility (Hoffmann *et al.* 1986; Giordano *et al.* 1995; Bourtzis *et al.* 1996; Charlat *et al.* 2002; Richardson *et al.* 2016). However, in many cases Wolbachia does not appear to behave as a reproductive parasite in Drosophila. Strains that induce neither cytoplasmic incompatibility nor male-killing have been identified in a number of species (Hoffmann *et al.* 1996; Zabalou *et al.* 2004; Hamm *et al.* 2014).

In some cases Wolbachia infection appears to have a fitness benefit to the host. These fitness benefits largely fall into two categories: survival/reproduction and immune-defense. With respect to the former, several studies in *D. melanogaster* have revealed that Wolbachia-infected flies can enjoy enhanced survival and fecundity (Fry and Rand 2002; Fry *et al.* 2004; Serga *et al.* 2014). These fitness benefits could contribute to the high Wolbachia infection rates seen in natural populations of *D. melanogaster* (Serga *et al.* 2014). With regard to immune-related benefits, Wolbachia infection confers protection against viral infection in Drosophila, leading to reduced viral load and/or decreased mortality associated with viral infection (Hedges *et al.* 2008; Teixeira *et al.* 2008; Osborne *et al.* 2012; Chrostek *et al.* 2013; Stevanovic *et al.* 2015). Data on whether Wolbachia infection confers resistance or tolerance to bacterial infection are less clear. Although some studies have shown no Wolbachia-mediated antibacterial protection in *D. melanogaster* (Wong *et al.* 2011; Rottschaefer and Lazzaro 2012; Shokal *et al.* 2016), other data are suggestive of a protective effect of Wolbachia infection against secondary infection with pathogenic bacteria (Unckless *et al.* 2015 though we note that the effects of host genotype cannot be ruled out). It has recently been suggested that the antibacterial protective effects of Wolbachia infection may be tied to whether the secondary infection is enteric or systemic, with protective properties evident in the case of enteric but not systemic infections (Gupta *et al.* 2017).

The demonstrated effects of Wolbachia on both reproductive and immune phenotypes in Drosophila led us to consider the potential effects of Wolbachia on meiotic recombination rate. Recombination rate has been shown to exhibit phenotypic plasticity in Drosophila in response to a variety of stressors including temperature (Plough 1917; Plough 1921; Stern 1926; Smith 1936; Grushko *et al.* 1991) and maternal age (Stern 1926; Bridges 1927; Redfield 1964; Lake 1984; Chadov *et al.* 2000; Priest *et al.* 2007; Tedman-Aucoin and Agrawal 2012; Hunter *et al.* 2016b). Plastic recombination has also been shown in Drosophila in response to stressors such as heat shock (Jackson *et al.* 2015) and starvation (Neel 1941).

We have recently shown that *D. melanogaster* females plastically increase their recombination fraction in response to infection with a pathogenic bacterium (Singh *et al.* 2015). This is consistent with a growing body of literature indicating a connection between stress and increased recombination. Here we test the extent to which bacterial infection with Wolbachia alters the recombination fraction in *D. melanogaster* females. Unlike previous stimuli surveyed, Wolbachia infection is not generally considered as a ‘stressor.’ Rather, the prevalence of Wolbachia infection in nature coupled with the strictly vertical transmission may have instead led to a mutually beneficial relationship between host and endosymbiont. It was thus unclear whether Wolbachia would indeed affect recombination in *D. melanogaster* females. However, meiotic recombination occurs during oogenesis and Wolbachia colonize the female germline, setting the stage for a potential link between Wolbachia infection and recombination.

To test for an effect of Wolbachia infection on recombination rate in *D. melanogaster* females, we surveyed recombination using a classical genetic approach in wild-derived Wolbachia-infected and uninfected strains, controlling for genotype. We measured recombination in two genomic intervals—one autosomal and one X-linked. Our data indicate that recombination rates are significantly higher in Wolbachia-infected flies as compared to their genetically matched uninfected controls, but only for one of the two intervals surveyed. These data are particularly interesting because the strain of Wolbachia in the current experiment is not considered to be pathogenic. This is thus a departure from previous work, which has focused primarily on stressful conditions, and may point to a more general connection between bacterial infection and recombination rate. Our data are also suggestive that although Wolbachia infection does not significantly affect the sex ratio of progeny produced, it does yield increased reproductive output for Wolbachia-infected females relative to uninfected females. Given the high frequencies of infection by Wolbachia in natural populations *of D. melanogaster*, our results suggest that variation in recombination rate and reproductive output of *D. melanogaster* females in nature may be driven in part by Wolbachia.

## Methods

### Stocks and fly rearing

The eight wild-type lines used for this experiment were selected from the Drosophila Genetic Reference Panel (Mackay *et al.* 2012). These lines are: RAL149, RAL306, RAL321, RAL365, RAL790, RAL853, RAL879, and RAL897. All of these strains have standard chromosome arrangements. These lines are naturally infected with *Wolbachia pipientis* (Huang *et al.* 2014). Genomic analysis indicates that the colonizing Wolbachia strain is a *wMel*-like strain (Richardson *et al.* 2012). *wMel* has been shown to induce cytoplasmic incompatibility, though the magnitude of the effect varies among studies (Bourtzis *et al.* 1996; Poinsot *et al.* 1998; Mcgraw *et al.* 2002; Reynolds and Hoffmann 2002; Yamada *et al.* 2007). We created Wolbachia-free versions of these eight strains. To cure the strains of Wolbachia infection, we raised flies on tetracycline-containing media for two consecutive generations. These flies were raised in 8 ounce (oz) bottles with a standard cornmeal/molasses media containing tetracycline (dissolved in ethanol) to a final concentration of 0.25 mg/ml media. After the second generation of tetracycline treatment, these strains (denoted, for example, RAL149*^w^*^-^) were raised on standard media for more than five generations before the experiment described below began.

We used doubly-marked strains for our estimation of recombination rate. The markers used to measure recombination on the X chromosome were *yellow* (*y*^1^) and *vermilion* (*v*^1^) (Bloomington Drosophila Stock Center #1509), which are 33 cM apart (Lindsley and Grell 1967). We integrated this doubly-marked X chromosome into the wild-type isogenic Samarkand genetic background (Lyman *et al.* 1996); this line abbreviated hereafter as ‘*y v*’. The markers on the chromosome 3R were *ebony* (*e*^4^) and *rough* (*ro*^1^) (Bloomington Drosophila Stock Center #496), which are 20.4 cM apart (Lindsley and Grell 1967); this line is abbreviated hereafter as ‘*e ro*.’ These markers and strains have been used extensively in our lab to estimate recombination frequency (Jackson *et al.* 2015; Singh *et al.* 2015; Hunter *et al.* 2016a; Hunter *et al.* 2016b). Both of the marker strains have a standard chromosome arrangement.

### Wolbachia screen

Immediately prior to conducting these experiments, we confirmed the presence of Wolbachia infection in the standard RAL lines (denoted, for example, RAL149*^w^*^+^) and the absence of Wolbachia in the tetracycline-treated lines using a PCR-based assay with Wolbachia-specific primers. Four adult females were used per line to test for Wolbachia infection. Briefly, DNA was extracted from each female using a standard squish prep (Gloor and Engels 1992). Each fly was crushed with a motorized pestle and subsequently immersed in a buffered solution (10 mM Tris-Cl pH 8.2, 1 mM EDTA, 25 mM NaCl, 200 μg/ml proteinase K). This was incubated at 37° for 30 min and then at 95° for 2 min to inactivate the proteinase K. We used Wolbachia-specific primers wspF and wspR (Jeyaprakash and Hoy 2000) to test for presence/absence of Wolbachia infection.

Amplifying conditions were as follows: 94°/3 min, 12 cycles of 94°/30 sec, 65°/30 sec, 72°/60 sec with the annealing temperature reduced by 1.0 degrees per cycle, followed by 25 cycles of 94°/30 sec, 55°/30 sec, 72°/60 sec. We included a final extension of 72°/7 min. All PCR reactions were 10 μl, and each contained 5 μl Qiagen 2X PCR Master- Mix, 0.25 μl of each 20 mM primer, 3.5 μl H_2_O, and 1 μl genomic DNA. All four tested females from each of the eight Wolbachia-infected lines showed evidence of Wolbachia infection using this assay, while none of the females from the tetracycline-treated lines showed any evidence for Wolbachia infection.

### Experimental crosses

To assay recombination rate variation in the experimental lines, we used a classic two-step backcrossing scheme. All crosses were executed at 25° with a 12:12 hr light:dark cycle on standard media using virgin females aged roughly 24 hr. For the first cross, ten virgin females from each experimental evolution line were crossed to ten *e ro* (or *y v*) males in 8 oz bottles. Males and females were allowed to mate for five days, after which all adults were cleared from the bottles. F_1_ females resulting from this cross are doubly heterozygous; these females are the individuals in which recombination is occurring. To uncover these recombination events we backcrossed F_1_ females to doubly-marked males. For this second cross, twenty heterozygous virgin females were collected and backcrossed to twenty doubly-marked males in 8 oz bottles. Males and females were allowed to mate for five days, after which all adults were cleared from the bottles. After eighteen days, BC_1_ progeny were collected and scored for sex and for visible phenotypes. Recombinant progeny were then identified as having only one visible marker (*e*+ or +*ro* in the case of crosses involving the *e ro* double mutant, or *y*+ or +*v* in the crosses involving the *y v* double mutant). Ten to fifteen replicate (second) crosses were set up for each strain. For each replicate, recombination rate was estimated by taking the ratio of recombinant progeny to the total number of progeny. Double crossovers cannot be recovered with this assay, so our estimates of recombination frequency are likely to be biased downward slightly.

### Statistical analyses

All statistics were conducted using JMPPro v13.0. To test for factors associated with variation in recombination fraction, we used a generalized linear model with a binomial distribution and logit link function on the proportion of progeny that is recombinant. We treated each offspring as a realization of a binomial process (either recombinant or nonrecombinant), summarized the data for a given vial by the number of recombinants and the number of trials (total number of progeny per vial), and tested for an effect of line and Wolbachia status plus the interaction of line and Wolbachia status. We note that ‘line’ incorporates known differences between host genotypes and potential unknown differences in Wolbachia genotype. This logistic regression approach thus takes the total number of observations per vial into account (giving more weight to vials with more progeny, where the estimation of recombination is likely to be more accurate). The full model is as follows:

$${\text{Y}}_{\text{ij}}=\mu +L_{\text{i}}+W_{\text{j}}+L\text{xW }+\text{ε}$$

for:i= 1...8,j= 1...2

*Y* represents the proportion of progeny that is recombinant, μ represents the mean of regression, and ε represents the error. *L* denotes strain, *W* denotes Wolbachia infection status, and *L*x*W* denotes the interaction of line and Wolbachia infection status. All of these are modeled as fixed effects.

To test for potential differences between the two different intervals surveyed, we employed a similar logistic approach. The full model is as follows:

$$Y_{\text{ij}}=\text{μ}+L_{\text{i}}+W_{j}+{\text{ I}}_{k}+L\text{xW }+L\text{xI }+I\text{x}W\;+L\text{x}W\text{xI }+\text{ε}$$

for:i= 1...8,j= 1...2, k=1…2

*Y* represents the proportion of progeny that is recombinant, μ represents the mean of regression, and ε represents the error. *L* denotes strain, *W* denotes Wolbachia infection status, and *I* denotes interval surveyed. There are four interaction terms: *L*x*W* denotes the interaction of line and Wolbachia infection status, *L*xI is the interaction between line and interval, *I*xW is the interaction between interval and Wolbachia infection status, and *L*xWxI is the interaction among line, Wolbachia infection status and interval. All of these are modeled as fixed effects.

We note that whether ‘line’ could in principle be modeled as a fixed effect or a random effect, the choice of which to employ depends on the specific question that one is posing. If we wished to use the lines in the current experiment to say something about populations of *D. melanogaster* broadly speaking, then should model ‘line’ as a random effect. If instead we merely wish to interrogate this specific set of lines, not representative of any population, then modeling ‘line’ as a fixed effect is statistically appropriate. Our results speak to this set of lines alone, and we thus model line as a fixed effect. We have modeled ‘line’ as a fixed effect for similar questions in previous work (Hunter and Singh 2014; Hunter *et al.* 2016a; Hunter *et al.* 2016b; Kohl and Singh 2018).

To test for factors associated with variation in the sex ratio, we used the same generalized linear model framework with a binomial distribution and logit link function on the proportion of total number of progeny that is male. For each interval, the model is the same as is described above, except that *Y* represents the proportion of progeny that is male. We note that this analysis is independent of recombination frequency.

To test for factors associated with variation in reproductive output, we used an ANOVA framework. The ANOVA followed the form of *Y* = *μ* + *L* + W + *ϵ*, for each interval assayed where *Y* is reproductive output, *μ* is the overall mean, *L* is the fixed effect of line, *W* is the fixed effect of Wolbachia status, and *ϵ* is the residual.

### Availability of data and material

Strains are available upon request. Raw data have been deposited into Dryad (doi:10.5061/dryad.16dt35k).

## Results

### Viability effects of markers on recombination rate estimation

In total, 76,211 BC1 progeny were scored for recombination for the *e ro* interval, and 79,447 flies were scored for recombination in the *y v* interval. For the *e ro* interval, the number of progeny per bottle ranged from 123-732, with an average of 476 BC1 progeny per vial. For the *y v* interval, the number of BC1 progeny ranged from 230-630, with an average of 462 progeny per bottle.

To test for deviations from expected ratios of phenotype classes, we performed *G*-tests for goodness of fit for all crosses for the following ratios: males *vs.* females, wild-type flies vs. e ro (or y v) flies and finally, *e* + flies *vs.* + *ro* flies (or *y* + *vs.* + *v* flies). The null hypothesis for each comparison is a 1:1 ratio of phenotype classes. We tested for such deviations within each bottle. Deviations from these expected ratios could reflect viability defects associated with the marked chromosomes, which could adversely affect our estimation of recombination rate.

Consistent with previous studies involving these strains (Hunter and Singh 2014, Hunter *et al.* 2016a, Hunter *et al.* 2016b), our data (see Supplemental information) suggest that there are small viability defects associated with the doubly marked chromosomes. However, these defects are not systematically biasing the estimates of recombination with respect to Wolbachia status in this experiment. That is, these skewed ratios do not appear depend on Wolbachia infection status. The full distributions of these ratios are plotted in Supplemental Figures 1 and 2. Fitting a generalized linear model with a binomial distribution and logit link function on the proportion of non-recombinant progeny that is wild-type shows no significant effect of Wolbachia status (*P* = 0.07, χ^2^ test (N = 172, df = 7)) for the *y v* crosses. The same result is found with the *e ro* crosses (*P* = 0.42, χ^2^ test (N = 160, df = 7)). We thus believe that the small viability defects associated with the doubly marked chromosome are not systematically biasing the estimates of recombination with respect to Wolbachia status in this experiment.

### Effects of Wolbachia infection status on recombination

**Figure 1:**
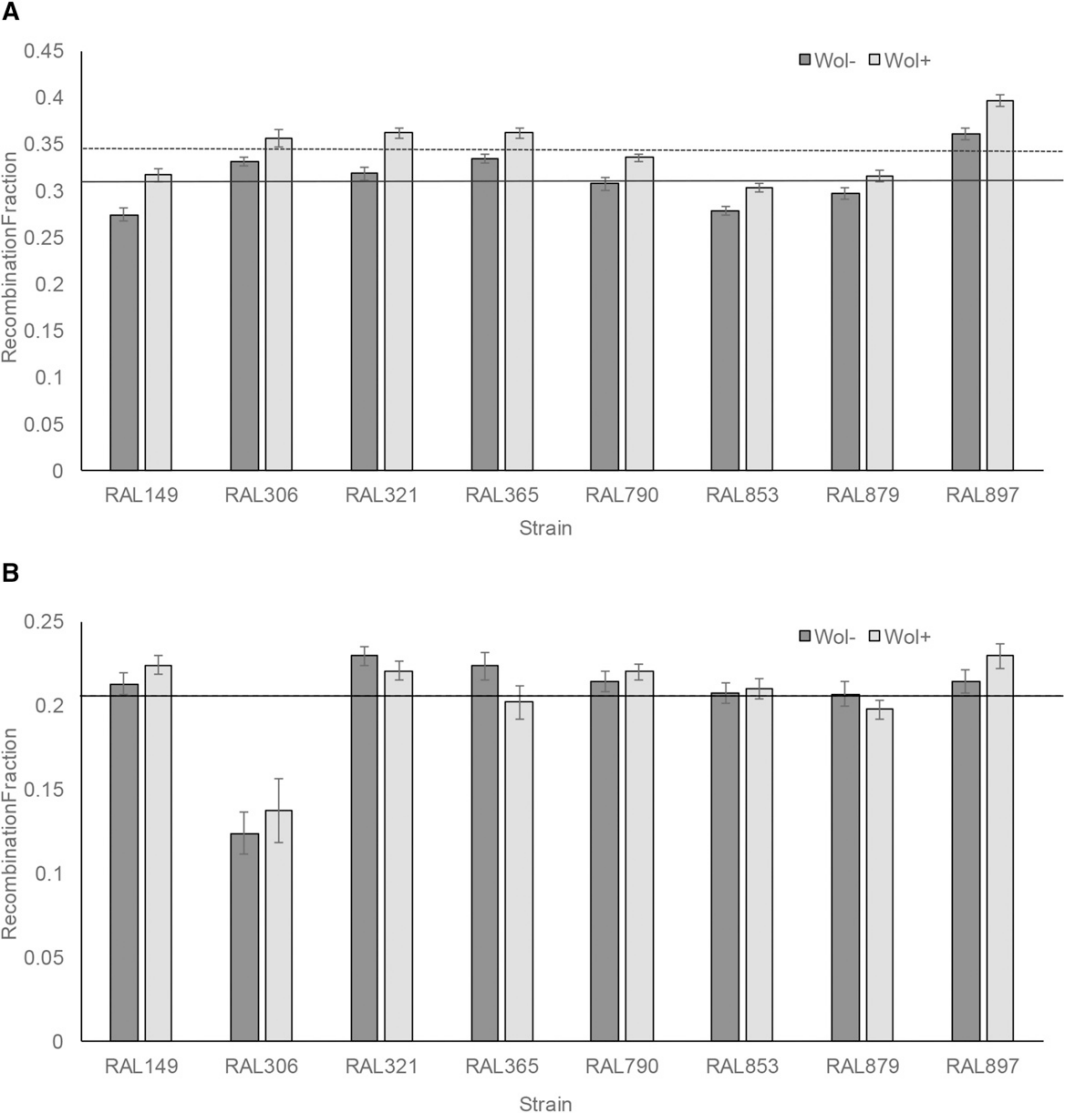
Mean recombination fraction between a) *yellow* and *vermillion* and b) *ebony* and *rough* interval as a function of genetic background and Wolbachia infection status. Dark gray bars are Wolbachia-free lines and the light gray bars are the Wolbachia-infected counterparts. Error bars denote standard error. Average recombination rate (across lines) is depicted for Wolbachia-infected (dashed line) and uninfected lines (solid line) though they are not both visible in panel b because they are so close together (0.204(uninfected) *vs.* 0.205(infected)).

We used a logistic regression model to identify factors significantly contributing to variation in recombination fraction observed in the current experiment. Note that because we are assaying recombination in heterozygous females (see Materials and Methods), we can only detect dominant genetic effects. The logistic regression model indicates that both genotype and Wolbachia infection status significantly contribute to variation in recombination rate in the *y v* interval (*P* < 0.0001, both factors, χ^2^ test (N = 172, df = 7 for genotype, df = 1 for Wolbachia status)). We remind the reader that ‘genotype’ conflates known differences in host genotype with potential differences in Wolbachia genotype. Average recombination values for each strain are presented in Figure 1, and these data illustrate that recombination fraction increases with Wolbachia infection. There is no significant interaction effect between Wolbachia status and genotype (*P* = 0.54, χ^2^ test (N = 172, df = 7)).

Different results are found for the *e ro* interval. Although genotype significantly contributes to variation in recombination fraction (*P* < 0.0001, χ^2^ test (N = 160, df = 7)), Wolbachia status does not (*P* = 0.31, χ^2^ test (N = 160, df = 1)). The lack of consistent effect of Wolbachia status on recombination fraction is echoed in Figure 1. There is a marginally significant interaction effect between Wolbachia status and genotype on recombination fraction (*P* = 0.04, χ^2^ test (N = 160, df = 7)). However, the distributions of recombination fractions for a given genotype with Wolbachia and without Wolbachia do not differ significantly in any of the eight comparisons (*P* > 0.08, all tests, Wilcoxon rank test).

To test explicitly for a difference between the two intervals with regard to the effect of Wolbachia on recombination, we again rely on logistic regression (see Methods). This analysis indicates significant effects of line (*P* < 0.0001, χ^2^ test (N = 332, df = 7)) and Wolbachia infection status (*P* < 0.0001, χ^2^ test (N = 332, df = 1)). We also find a significant effect of interval (*P* < 0.0001, χ^2^ test (N = 332, df = 1)), consistent with differences in underlying map distance between the two intervals. Importantly, we find a significant interaction effect of interval and Wolbachia infection status (*P* < 0.0001, χ^2^ test (N = 332, df = 1)), which indicates that the effect of Wolbachia infection status on recombination fraction is significantly different between the two intervals. The interval by line interaction is significant (*P* < 0.0001, χ^2^ test (N = 332, df = 7)) though the three-way interaction among line, interval and Wolbachia infection status is not (*P* = 0.18, χ^2^ test (N = 332, df = 7)).

### Effects of Wolbachia infection status on other phenotypes

**Figure 2:**
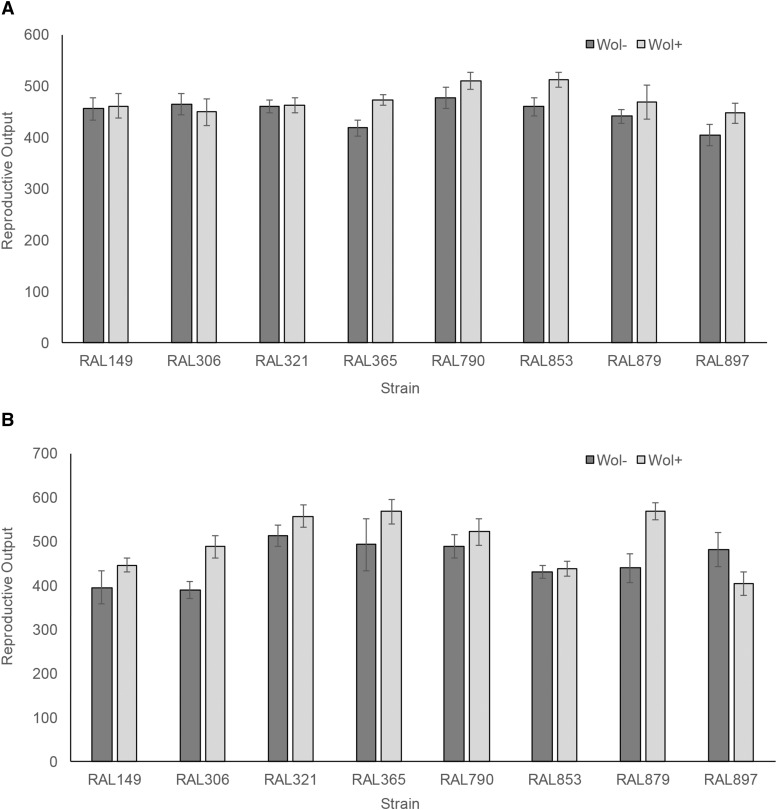
Mean reproductive output for crosses measuring recombination in the a) *yellow* and *vermillion* and b) *ebony* and *rough* interval as a function of genetic background and Wolbachia infection status. Dark gray bars are Wolbachia-free lines and the light gray bars are the Wolbachia-infected counterparts. Error bars denote standard error.

Given the known effects of Wolbachia infection on host biology, we tested for effects of Wolbachia infection on two additional phenotypes: reproductive output and sex ratio. To examine factors associated with variation in reproductive output, we used an ANOVA framework. For the crosses involving *y v* flies, both genotype and Wolbachia status significantly affect reproductive output (as measured by the number of progeny in a replicate) (*P*_Genotype_ = 0.03, *P*_Wolbachia_ = 0.01, respectively). Figure 2 illustrates that reproductive output generally increases with Wolbachia infection. There is no significant interaction between genotype and Wolbachia status (*P* = 0.63). For the crosses involving *e ro* flies, genotype and Wolbachia status again contribute to reproductive output (*P*_Genotype_ < 0.0001, *P*_Wolbachia_ = 0.003, respectively); Wolbachia infection results in an increase in reproductive output (Figure 2b). For these crosses, there is also a significant interaction effect between Wolbachia status and genotype (*P* = 0.03), with Wolbachia status impacting reproductive output in some strains more strongly than others (Figure 2b). Again, effects of ‘genotype’ may be due to host genetic differences or yet unknown differences in Wolbachia substrains that may have differentially infected these strains.

To test for factors affecting the sex ratio, we used a logistic regression (see Materials and Methods). For the crosses involving *y v* flies, there are no significant effects of genotype, Wolbachia status, or the interaction between genotype and Wolbachia status on the ratio of males to females (*P* = 0.08, 0.55, and 0.48, respectively). Progeny resulting from backcrosses with *e ro* flies show the same result, with no significant contribution of genotype, Wolbachia status, or the interaction between genotype and Wolbachia status to the sex ratio (*P* = 0.10, 0.19, and 0.16, respectively).

## Discussion

Here we show that infection with Wolbachia significantly increases the recombination fraction for an X-linked genomic interval but not an autosomal region (Figure 1). Previous work with this experimental framework revealed that Wolbachia infection had no detectable effect of levels of crossing over in *D. melanogaster* (Serga *et al.* 2010). The discrepancy between that finding and the current study may be due to the fact that different *D. melanogaster* genotypes were interrogated in the two studies. There may be underlying differences in the Wolbachia genomes infecting the host strains between the two studies as well. In addition, different genomic intervals were surveyed in the two studies (though we note that our X-linked locus encompasses that utilized in the initial study). Indeed, the degree to which recombination is plastic varies among genotypes and among loci in *D. melanogaster* (Hunter *et al.* 2016b).

That one genomic interval shows a plastic response to Wolbachia infection while another does not is also consistent with previous reports indicating variation in recombination plasticity across intervals in *D. melanogaster* (Hunter *et al.* 2016b) and *D. pseudoobscura* (Stevison *et al.* 2017). Interestingly, the *e ro* interval surveyed here has previously been shown to exhibit a plastic increase in recombination following infection with pathogenic bacteria (Singh *et al.* 2015) and heat shock (Jackson *et al.* 2015), but no plastic response to maternal age (Hunter *et al.* 2016b). This suggests that genomic intervals may exhibit phenotypic plasticity in recombination in response to some environmental cues but not others. That infection with a bacterial endosymbiont does not elicit a response in the *e ro* interval while infection with a pathogenic bacterium does could indicate that the mechanisms by which the recombination fraction is increased in differs between these two types of bacteria. The *y v* interval surveyed in the current study, showing an increase in recombination associated with Wolbachia infection also shows an increase in recombination with maternal age (Hunter *et al.* 2016b). Because the four of five lines studied in the maternal age study were also Wolbachia-infected, it is formally possibly that maternal age and Wolbachia infection maybe be linked in some way. Perhaps Wolbachia titer changes over time, or perhaps Wolbachia infection affects developmental rates. That notwithstanding, it remains to be determined what causes some loci to exhibit plastic recombination while others do not, and why some loci show plastic recombination under certain conditions but not others.

That Wolbachia affects recombination in an X-linked interval but not an autosomal one is potentially of interest. Because Wolbachia has been shown to affect the sex ratio in many systems (though not in the current study), there may be some interaction between Wolbachia and the X chromosome. Future work surveying recombination in additional sex-linked and autosomal regions is required to determine whether the observations in the current study are reflective of broader patterns. Future work could also include cytological assays to determine whether the X undergoes more meiotic double-stranded breaks in Wolbachia-infected *vs.* uninfected flies, and if autosomal breaks change in frequency in a Wolbachia-dependent way.

Recombination plasticity associated with bacterial infection has been previously shown in *D. melanogaster* as well (Singh *et al.* 2015). In contrast to the pathogenic bacteria used in that experiment (*Serratia marcescens* and *Providencia rettgeri*), the *wMel* strain of *Wolbachia pipentis* used in the current experiment is viewed more as a mutualistic endosymbiont rather than a pathogenic bacterium. That a stably co-evolving, vertically transmitted endosymbiont can elicit significant effects on recombination rate is both surprising and exciting.

The aforementioned plastic increase in recombination associated with bacterial infection with pathogenic bacteria appeared to be driven in part by an active immune response (Singh *et al.* 2015). Is Wolbachia infection associated with immune signaling? Several studies have indicated that the *D. melanogaster* transcriptional profile significantly differs between Wolbachia-infected and Wolbachia-free flies. *In vitro* examination of S2 cells challenged with Wolbachia showed upregulation of several immune genes including *Toll*, *Imd*, and five antimicrobial peptides (Xi *et al.* 2008). Similarly, larval testes show significant upregulation of nine immune-related genes in Wolbachia-infected males compared with uninfected controls (Zheng *et al.* 2011). However, other results show no effect of Wolbachia alone on regulation of immune genes (Bourtzis *et al.* 2000; Wong *et al.* 2011; Shokal *et al.* 2016). Future work includes comparing expression profiles of genotype-controlled Wolbachia-infected and Wolbachia-free flies to explicitly test for differences in differences in expression for genes in the innate immune pathway.

In addition to potentially affecting immune signaling, Wolbachia infection has been associated with key changes in germline function. This is of importance to our study given its focus on meiotic recombination, which occurs in the female germline. This has been demonstrated in both *D. melanogaster* and *Drosophila mauritiana* (Fast *et al.* 2011; Touret *et al.* 2014; Christensen *et al.* 2016). Wolbachia infection also increases the frequency of apoptosis in oogenesis in both mosquitoes (Almeida and Suesdek 2017) and *D. melanogaster* (Zhukova and Kiseleva 2012). This is particularly intriguing; the stage of oogenesis that was examined and showed higher levels of apoptosis associated with Wolbachia infection was region 2a/2b of the germarium. This is precisely where double strand breaks are initiated and resolved (Jang *et al.* 2003; Staeva-Vieira *et al.* 2003; Gorski *et al.* 2004). This suggests that Wolbachia infection has the capacity to affect host reproductive phenotypes in exactly the region in which crossovers are formed.

Consistent with its effect of on the female germline, our data further indicate that Wolbachia infection significantly increases reproductive output (Figure 2), at least as measured in our experimental framework. This echoes what has been shown in *D. simulans* (Weeks *et al.* 2007), *D. mauritiana* (Fast *et al.* 2011) and *D. suzukii* (Mazzetto *et al.* 2015 though see also Hamm *et al.* 2014). In *D. melanogaster*, females infected with mutualistic strain *wMel* (denoted wDm in some early literature) generally show higher reproductive output than Wolbachia-uninfected flies (Fry *et al.* 2004; Serga *et al.* 2014). This gives some credence to the idea that infections with *wMel* benefit the host, and that these benefits may have evolved from the stability of the host-endosymbiont relationship given vertical transmission.

Unlike reproductive output, the sex ratio of progeny produced did not differ systematically between Wolbachia-infected and uninfected flies in the current study. This mirrors previous results in *D. melanogaster* (with or without wMel), which also showed no significant difference in sex ratio associated with Wolbachia status (O’shea and Singh 2015; Ponton *et al.* 2015). Although Wolbachia strains that manipulate the sex ratio clearly exist in other Drosophila species (Hurst *et al.* 2000; Dyer *et al.* 2005; Sheeley and Mcallister 2009; Richardson *et al.* 2016), wMel does not appear to have a feminizing effect in *D. melanogaster*. However, because the environment can shape the effects of Wolbachia on its host, it remains possible that wMel could shift the sex ratio in *D. melanogaster* under different environmental conditions.

## Conclusions

Recombination frequency is phenotypically plastic in a number of organisms. This is perhaps best studied in *Drosophila melanogaster*, where the first reports of plastic recombination were published nearly a century ago. Here we test the hypothesis that Wolbachia infection increases recombination in *D. melanogaster*. By surveying recombination at two loci in eight wild-derived strains of *D. melanogaster*, we show that infection with Wolbachia is associated with increased recombination in one genomic interval but not another interval. It is not known whether this effect is modulated by the immune system–this is a topic of future work. The factors underlying the genomic heterogeneity in the plastic recombinational response are unknown as well. We further find that reproductive output is increased with Wolbachia infection, suggestive of a benefit of infection to the host in our experimental framework. Given that Wolbachia are maternally-transmitted, increased reproductive output of the fly host boosts not only host reproductive fitness, but the fitness of Wolbachia as well. Given the prevalence of Wolbachia infection in natural populations of Drosophila, these findings markedly impact our understanding of natural variation in recombination and reproduction in natural populations of *D. melanogaster*.
